# Intrafetal laser therapy in a monochorionic diamniotic triplet pregnancy with two acardiac fetuses: a case report and literature review

**DOI:** 10.1186/s12884-021-03716-6

**Published:** 2021-03-23

**Authors:** Guiqiong Huang, Hua Liao, Qing Hu, Xiaodong Wang, Haiyan Yu

**Affiliations:** 1 Department of Obstetrics and Gynecology, West China Second University Hospital, Sichuan University, No. 20, 3rd section, South Renmin Road, Sichuan 610041 Chengdu, China; 2 Key Laboratory of Birth Defects and Related Diseases of Women and Children (Sichuan University), Ministry of Education, Chengdu, China

**Keywords:** Monochorionic diamniotic triplets, Twin reversed arterial perfusion sequence, Intrafetal laser therapy

## Abstract

**Background:**

Monochorionic diamniotic triplet pregnancies are rare. Twin reversed arterial perfusion sequence in monochorionic triplet pregnancies is extremely rare, and it is associated with high perinatal morbidity and mortality rates in the “pump fetus.”

**Case presentation:**

We reported a case of monochorionic diamniotic triplet pregnancy with twin reversed arterial perfusion sequence, including two acardiac fetuses sharing a single amniotic sac and a normal fetus in another amniotic sac. Due to rapid growth of the acardiac fetuses, intrafetal laser therapy was performed in both of them under ultrasound guidance at 15 weeks +5 days. Subsequently, regular and careful antenatal care including fetal ultrasonography and doppler and fetal echocardiography was conducted. At 37 weeks +4 days, a healthy female baby weighing 2510 g was delivered. The baby was followed up and now at 11 months old is in good health.

**Conclusions:**

Twin reversed arterial perfusion sequence in monochorionic triplet pregnancy should be diagnosed early by ultrasound imaging during pregnancy. Individualized management should be based on clinical conditions to improve the perinatal outcome of the pump twin. Intrafetal laser therapy could be an alternative procedure when intrauterine intervention is required.

## Background

The incidence of triplet pregnancies has increased significantly in recent decades due to the use of assisted reproductive technology and the trend of delayed childbearing, with the most common type being trichorionic triamniotic pregnancy [1, 2]. Monochorionic triplet pregnancies occur at a rate of approximately 1 in 45,500 deliveries [3]. Monochorionic diamniotic (MCDA) triplet pregnancies are exceedingly rare compared with other types of triplet pregnancies. There have been only a few published articles relevant to MCDA triplet pregnancies so far, and the majority of them are case reports [4–15]. Triplet pregnancies are associated with higher perinatal morbidity and mortality as well as life-threatening maternal complications [16], especially in MCDA triplet pregnancies due to their special chorionic and amniotic properties.

Twin reversed arterial perfusion (TRAP) sequence is a rare and specific complication of monochorionic multiple pregnancies. The incidence of TRAP in monochorionic triplet pregnancies is approximately 1 in 4.5 million pregnancies [17]. Here, we reported a successful application of intrafetal laser therapy in an extremely rare case of MCDA triplet pregnancy with two acardiac fetuses and successful delivery of a healthy newborn at term at the West China Second University Hospital, a tertiary referral center in western China. The treatment procedure followed ethical principles; all data were collected from chart reviews, and an approval was obtained from the Institutional Review Board. Additionally, we conducted a literature review about TRAP sequence in monochorionic triplet pregnancies.

## Case presentation

A 31-year-old woman, gravida 2 para 0, spontaneously conceived. Ultrasound examination at 12 weeks’ gestation confirmed monochorionic diamniotic triplets including two acardiac fetuses (Fetus 2 and Fetus 3) sharing a single amniotic sac and one normal fetus (“pump fetus” or Fetus 1) in another amniotic sac. The patient’s serology was negative for human immunodeficiency virus (HIV), venereal disease research laboratory (VDRL), and hepatitis B surface antigen (HBsAg), and she had no diabetes mellitus. The pregnant woman and her partner reported no history of medication, substance abuse, and family history of congenital anomalies. They were extensively counseled by the multidisciplinary team and the woman was followed up with serial fetal ultrasonography. Fetus 2 and Fetus 3 in one amniotic sac were malformed with dysplasia of the spine and lower limbs, and complete absence of the head, heart, and upper limbs.

**Fig. 1:**
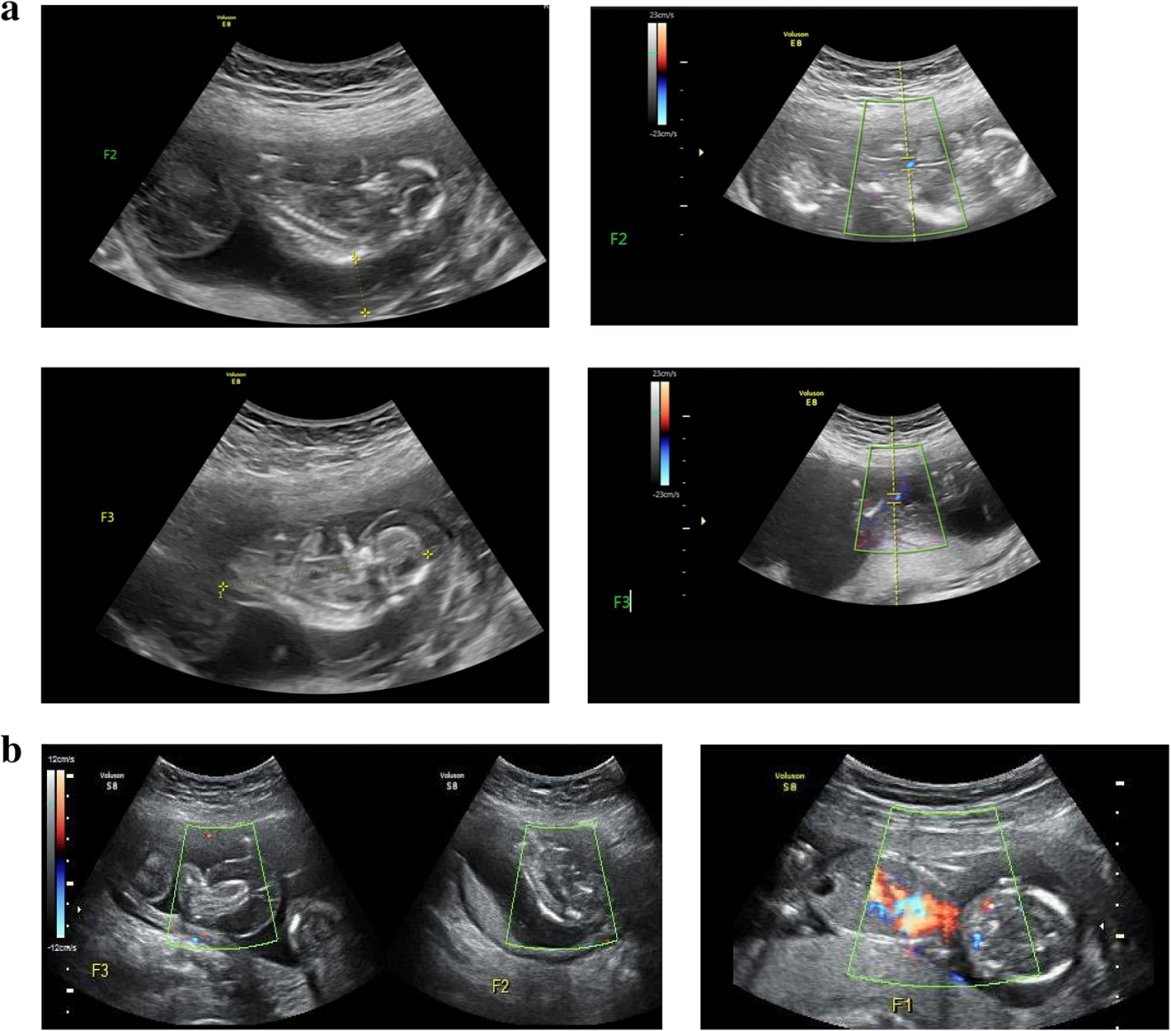
**a**. Images of increasing magnification show the acardiac fetuses (F2 and F3) and their reversed blood flow before intrafetal laser therapy. **b**. Images of pump fetus and both acardiac fetuses after intrafetal laser therapy

Due to rapid growth of the acardiac fetuses, after repeated extensive counseling about the predicted poor prognosis of the pump fetus (Fetus 1) during expectant management, intrafetal laser therapy was performed under ultrasound guidance at 15 weeks + 5 days. The18-gauge needle (Hakko Co., Ltd., Japan) was introduced into one of the acardiac fetuses. A 400-µm laser fiber was then passed through the needle and placed close to the pelvic vessels. Intrafetal laser therapy was performed using a NdYAG-laser source (Dornier MedTech, Wessling, Germany) in 5-s bursts at 10 W initial power, which was then doubled in steps to a maximum level of 20 W, thereby resulting in cessation of blood flow in that acardiac fetus. After that, the18-gauge needle was introduced into the other acardiac fetus, and the same procedure was done. Images of increasing magnification showing the acardiac fetuses (F2 and F3) and their reversed blood flow before intrafetal laser therapy are shown in Fig. 1a. Images of the pump fetus and both acardiac fetuses after intrafetal laser therapy are shown in Fig. 1b. The woman was followed up closely with fetal ultrasonography and doppler and fetal echocardiography.

The couple refused the chromosome examination in both acardiac fetuses, and amniocentesis was performed in the pump fetus. The result of chromosome microarray analysis in the pump fetus was normal.

**Fig. 2:**
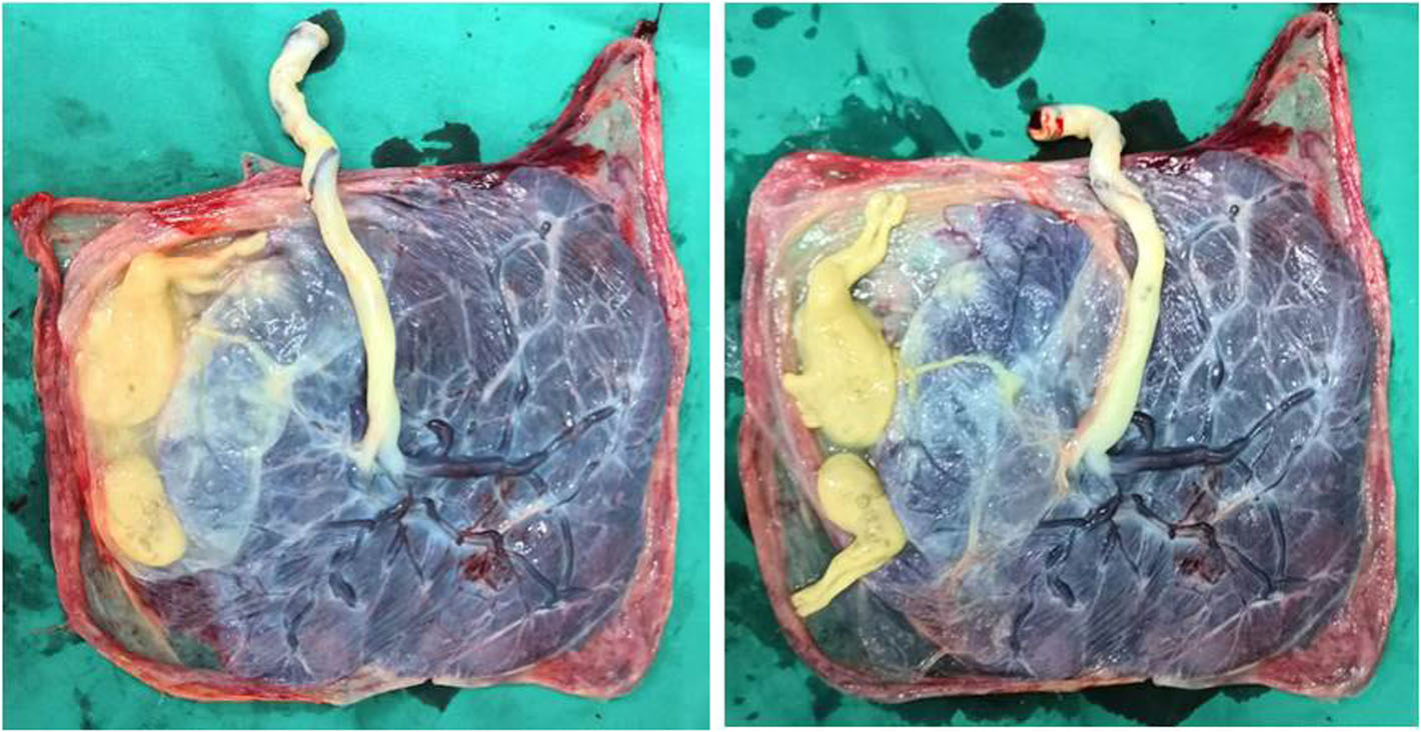
Images of the monochorionic-diamniotic placenta and the papyraceous acardiac fetuses

At 37 weeks + 4 days, a healthy female baby weighing 2510 g was delivered with Apgar scores of 10 and 10 at the first and fifth minute, respectively, whereas two papyraceous acardiac fetuses weighed 14 and 8 g. Images of the monochorionic-diamniotic placenta and the papyraceous acardiac fetuses are shown in Fig. 2. The pathological results revealed monochorionic-diamniotic triplet pregnancy with two acardiac fetuses. The baby is now 11 months old and she is in good health.

## Discussion and conclusions

TRAP sequence is characterized by the presence of an “acardiac twin” (with an absent or nonfunctioning heart) and a “pump twin” that provides perfusion that occurs exclusively in monochorionic multiple pregnancies. It is even more unusual in a triplet pregnancy. It is widely believed that the TRAP sequence is a result of abnormal vascular anastomoses between fetuses.

We used a list of keywords including “twin reversed arterial perfusion sequence,” “TRAP sequence,” “acardiac fetus,” “acardiac twin,” “triplets,” “triplet pregnancy,” and “multiple pregnancy” to perform an extensive search of literature in English and Chinese about the perinatal management and outcomes of monochorionic triplet pregnancies with TRAP sequence. The pump–acardiac complex in monochorionic triplets may present as one of the following three types: one acardiac with two pump fetuses; two acardiac fetuses with one pump fetus; one acardiac, one pump, and one unaffected fetus [17].

Our literature review revealed 17 published articles about monochorionic triplets with one acardiac fetus [17–33]. The management and perinatal outcomes of the acardiac fetus in monochorionic diamniotic triplet pregnancy and monochorionic triamniotic triplet pregnancy are shown in Table 1.

**Table 1:** Reported cases of monochorionic triplets with one acardiac fetus

Study ID	Maternal age (years)	Mode of conception	Triplet type	Diagnosis age(wks)	Prenatal intervention	Intervention weeks	Delivery age(wks)	Delivery method	Pregnancy outcomes
Kobori S 2019 [19]	38	NS	MCTA	10	RAF	18	37	CS	two male live infants weighing 2662 g and 2031 g, one acardiac fetus
Yldrm E 2019 [20]	30	spontaneously conceived	MCDA	31	No	/	35+4	CS	two live infants weighing 2010 g and 2150 g
Takano M 2019 [21]	36	spontaneously conceived	MCTA	16+1	Fetoscopic laser surgery	16+1	28+4	CS	two live infants weighing 917 g and 773 g
Yuan H 2017 [22]	39	IVF-ET	MCDA	10	No	/	early pregnancy	induced labor	one acardiac twin and conjoined pump twins
Li Q 2017 [23]	32	IVF-ET	MCDA	21	Intrathoracic selective reduction of the acardiac twin by injecting 15 mL of 10% NaCl	21+	35+4	CS	two male live infants weighing 2050 g and 1900 g
Takahashi Y 2018 [24]	29	NS	MCTA	13	RAF	16+3	29+3	CS	two female infants weighing 1167 g and 1237 g, one macerated acardiac fetus
Sugibayashi R 2016 [25]	NS	NS	MCTA	13+5	RAF	16+2	36+5	NS	two live infants weighing 2518 g and 2732 g, one acardiac fetus
Chaveeva P 2014 [18]	NS	NS	MCDA	14+5	No	/	22+2	Induced labor due to intrauterine demise of the two fetuses	two dead fetuses
	NS	NS	MCDA	16+2	No	/	17	Induced labor due to intrauterine demise of the two fetuses	two dead fetuses
	NS	NS	MCTA	24+3	No	/	34+1	NS	two live infants
	NS	NS	MCTA	13	Endoscopic laser	16	35+4	NS	two live infants
	NS	NS	MCTA	16	Intrafetal laser	16	37	NS	two live infants
López-Pérez R 2015 [26]	28	NS	MC triplet	20	No	/	32	CS	two live infants weighing 1821 g and 1512 g
Argoti PS 2013 [17]	22	NS	MCTA	21	RAF	23+4	31+5	CS	two live infants weighing 1515 g and 1275 g
Abi-Nader K 2009 [27]	25	spontaneously conceived	MCDA	15	No	/	26+5	CS	two live infants weighing 640 g and 1000 g, one acardiac fetus
Sepulveda1 W 2009 [28]	33	spontaneously conceived	MCDA	17	No	/	31	CS	two structurally normal infants weighing 1320 g and 1640 g, one acardiac fetus
	38	spontaneously conceived	MCTA	10	No	/	12	Dilatation and curettage due to intrauterine demise of the two fetuses	intrauterine demise of the two normal fetuses
Lee H 2007 [29]	NS	NS	MCDA	NS	RAF	NS	30	NS	two live infants
Van Schoubroeck D 2004 [30]	NS	NS	MCTA	13	Laser cord occlusion	16	38	NS	two live infants
	NS	NS	MCTA	13	Laser cord occlusion	19	36	NS	one live infant, one dead fetus
	NS	NS	MCTA	13	Laser cord occlusion	17	35	NS	two live infants
Dahiya P 2004 [31]	32	spontaneously conceived	MCDA	early second trimester	No	/	term	CS	two female live infants weighing 2300 g and 2500 g, one acardiac fetus
Pascal A 2000 [32]	20	spontaneously conceived	MCTA	17	No	/	17+	NS	two structurally normal fetuses weighing 149 g and 99g, one acardiac fetus
Bolaji II 1992 [33]	21	NS	MCTA	18	No	/	32	CS	two female live infants, one acardiac fetus

*NS* not specified, *IVF-ET* In vitro fertilization and embryo transfer, *MCTA* monochorionic triamniotic, *MCDA* monochorionic diamniotic, *MC triplet* monochorionic triplet, *wks* weeks, *RAF* radiofrequency ablation, *CS* cesarean section

To the best of our knowledge, there have been only three published articles in English and Chinese related to two acardiac fetuses with one pump fetus in a triplet gestation [4–6]. Sanjaghsaz et al. [5] first described the case of a pregnant woman who delivered a live female baby and conjoined acardiac acephalic twins by cesarean section at 35 weeks’ gestation. Ventura et al. [6] reported one case with two acardiac fetuses in monochorionic triamniotic triplet pregnancy. Spontaneous labor happened at 23 + 5 weeks, and there was no fetal heart activity in the pump twin. Furthermore, May et al. [4] reported a case of TRAP sequence in a MCDA triplet pregnancy with two conjoined acardiac fetuses. A live healthy male infant weighing 1275 g and two conjoined acardiac fetuses were delivered at 27 + 6 weeks. Detailed information of these cases is shown in Table 2.

**Table 2:** The characteristics of triplets with two acardiac fetuses

Study ID	Maternal age(years)	Spontaneously conceived	Triplet type	Diagnosis age(wks)	Prenatal intervention	Delivery age(wks)	Delivery method	Pregnancy outcomes
Sanjaghsaz 1998 [5]	24	NS	NS	At delivery	No	35 + 1	CS	A live infant weighing 2646 g and conjoined acardiac acephalic twins weighing 1350 g
Ventura 2011 [6]	30	spontaneously conceived	MCTA	21	No	23 + 5	VD	Spontaneous labor and no fetal heart activity in the pump twin, pump twin weighing 720 g, acardiac twins weighing 862 g
May 2016 [4]	30	NS	MCDA	26 + 1	No	27 + 6	CS	A live infant weighing 1275 g and conjoined acardiac twins weighing 940 g
Our case	31	spontaneously conceived	MCDA	12	Intrafetal laser therapy	37 + 4	CS	A healthy infant weighing 2510 g and two papyraceous acardiac fetuses weighing 14 and 8 g

*NS* not specified, *MCTA* monochorionic triamniotic, *MCDA* monochorionic diamniotic, *wks *weeks, *VD* vaginal delivery, *CS* cesarean section

With regard to the intrauterine intervention in multiple pregnancies with TRAP sequence, more studies have been conducted in twin than in triple pregnancies. The most widely used techniques in the management of TRAP pregnancies include ultrasound-guided ablation of intrafetal vessels by laser or radiofrequency (RFA) and bipolar cord coagulation because they are less invasive than endoscopic procedures [18]. Based on the literature [34], neonatal survival rate is comparable between RFA and intrafetal laser techniques (85 vs. 82 %, *P* = 0.63), but the incidence of premature rupture of membranes before 32 weeks’ gestation is significantly higher with RFA (22 vs. 7 %, *P* = 0.045).

With the widespread development of color doppler ultrasound, TRAP sequence can be diagnosed at 11–13 weeks of gestation. An early diagnosis allows accurate determination of chorionicity and also prompts for closer surveillance of the pregnancy, with earlier opportunity for intrauterine intervention to improve the survival rate of the pump fetus. It has been shown that adverse pregnancy outcomes are significantly less frequent when intrafetal laser treatment is undertaken before 16 weeks (3/16, 19 %) compared with at or after 16 weeks (19 % vs. 66 %; *P* = 0.0025) [34]. Chaveeva et al. [18] found a significant inverse association between gestational age at treatment and gestational age at birth (*r* = -0.297, *P* = 0.007). The mean gestational age at birth was 38 and 34 weeks when treatment was carried out at 13 and 27 weeks, respectively. Intrafetal laser therapy was suggested at 12–14 weeks rather than delaying intervention until 16–18 weeks. Paganie et al. [34] suggested that it might be advisable to restrict the administration of intrafetal laser treatment to a therapeutic window between 13 and 16 weeks of gestation. However, there is no consensus on management of the TRAP sequence.

Here we reported the application of intrafetal laser therapy in the MCDA triplet pregnancy complicated by two acardiac fetuses. We chose intrafetal laser as the best option at 15 weeks + 5 days to cease blood flow in both acardiac fetuses. After the procedure, the woman was stable throughout the pregnancy without complications. A full term newborn in good condition was finally delivered.

In conclusion, due to the rarity of triplet pregnancies with TRAP sequence, the experience with treatment is limited. Obstetricians and ultrasound specialists must be aware of the rare complication and focus on early ultrasound diagnosis. Individualized management should be based on fetal clinical conditions to improve the perinatal outcomes of the pump twin. Intrafetal laser therapy could be an alternative procedure when intrauterine intervention is required.

## Data Availability

The datasets used and/or analyzed during the current study are available from the corresponding author on reasonable request.

## Abbreviations

TRAP: Twin reversed arterial perfusion
MCDA: Monochorionic diamniotic
RFA: Radiofrequency ablation
